# Key questions to ask before implementing a Digital Mental Health Service (DMHS): A primer for policy makers^☆^

**DOI:** 10.1016/j.invent.2025.100857

**Published:** 2025-07-05

**Authors:** Nickolai Titov, Blake F. Dear, Lauren Staples, Alana Fisher, Heather D. Hadjistavropoulos, Olav Nielssen

**Affiliations:** a MindSpot Clinic, MQ Health, Macquarie University, NSW 2109, Australia; b School of Psychological Sciences, Macquarie University, NSW 2109, Australia; c Department of Psychology, University of Regina, Regina, Saskatchewan S4S 0A2, Canada

**Keywords:** Digital mental health services, Online mental health, E-mental health, Psychology services, Internet-delivered treatments, Implementation, Mental health policy, Mental health funding, Artificial intelligence, Virtual reality

## Abstract

**Background:**

The proven success of treating high prevalence mental disorders via the internet by digital mental health services (DMHSs) has created enormous interest in the implementation of these services. In response, there are now excellent guides to support the “how” of DMHS implementation.

**Method:**

Drawing on the authors' experiences of successfully implementing high volume DMHSs and reflecting on planning sessions with decision makers and funders, the authors identified important questions that should be considered by policy makers, funders and healthcare managers before implementing a DMHS. These questions are more concerned with the “why” of implementation and are questions not typically examined or discussed in existing implementation guides and frameworks.

**Results:**

The authors describe eleven questions categorised by theme: 1. The nature of mental health and treatments, 2. The nature of DMHSs, and 3. Governance and eco-system. Questions include which mental health conditions to address, whether the condition even requires treatment, what type of services should be offered, where would the DMHS fit into the broader mental health system, how will they integrate with other health services, what is the optimal funding model, how will they employ technology, and what governance is required.

**Conclusions:**

Policy makers and funders have the challenging task of determining resource allocation among competing priorities in a complex and ever-changing world. We propose that navigating the complexities of DMHSs can be facilitated by developing a robust program logic that addresses these and other important questions. It is noted that the long-term success DMHSs requires not only a clear vision and careful planning, but realistic and stable funding, and a commitment to ongoing evaluation and development.

## Highlights

• The proven success of digital mental health services has created enormous interest in “how” to implement these services.
• This review looks at “why” digital mental health services should be implemented.
• Policy makers and funders are encouraged to develop a clear program logic that answers these important questions.

## 1 Introduction

An increasing number of countries are funding the delivery of mental health services including educational materials, psychological assessments and psychological treatments via the internet (World Health Organization, 2022) using e-mental or digital mental health services (DMHSs). Systematic research reviews show that delivering psychological treatments online, for example, internet-delivered cognitive behavioural therapy (iCBT) guided by trained therapists, can achieve clinical outcomes comparable to face-to-face care (Hedman-Lagerlöf et al., 2023; Etzelmueller et al., 2020, and that these outcomes are replicated when such models are deployed with appropriate clinical governance in routine care (Hadjistavropoulos et al., 2011, Hadjistavropoulos et al., 2014; Titov et al., 2018, Titov et al., 2019; Vernmark et al., 2024).

As well as demonstrated effectiveness, factors driving interest in this model of service delivery include the positive experiences and preferences of consumers in accessing mental health services via technology (Jardine et al., 2024; Moskalenko et al., 2020), the increased availability of care to geographically isolated or marginalised consumers (Cuijpers et al., 2022; Staples et al., 2025), the availability of a cost-effective way of delivering services to a large number of people and recognition that existing in-person services cannot meet demand (Ftanou et al., 2024; Andersson and Berger, 2021; Andersson et al., 2019).

Numerous influential and helpful frameworks and guidelines have been developed to facilitate the implementation of interventions into health services (e.g., the Consolidated Framework for Implementation Research: CFIR; Damschroder et al., 2022), to support the assessment of the suitability of telemedicine applications for specific settings (for example, the Model for Assessment of Telemedicine Applications: MAST; Kidholm et al., 2012), and to optimise the implementation of DMHSs into mental health systems (Liu and Schueller, 2023; Mohr et al., 2025; Vis et al., 2023). Such frameworks provide robust evidence-based summaries and checklists typically focused on *how* to implement DMHSs, that is, the steps required to optimally and operationally deploy a DMHS. The purpose of the present paper is to examine and discuss important questions relating to both how and *why* to implement DMHSs. Some of these questions are addressed in existing frameworks, but some, for example, questions about the nature of mental health, are not typically included in existing frameworks or if they are, would benefit from further discussion of issues relevant to DMHSs for the benefit of policy makers and funders who are often less familiar with such questions.

The choice of these questions was informed by our experiences working with policy makers and funders when establishing and operating successful DMHSs in Australia and other countries (e.g., Dear et al., 2024a; Dear et al., 2024b; Fournier et al., 2025; Hadjistavropoulos et al., 2022; LeBlanc et al., 2022; Staples et al., 2022; Titov et al., 2020a; Titov et al., 2020b). The questions were identified by reviewing meeting notes held by the first author (NT) from July 2012 to December 2024 with >75 organisations from six countries who had sought advice about DMHSs. Several frequently asked questions about mental health and about DMHSs were identified from these notes and circulated among the authors. These questions were then categorised into three themes: 1. The nature of mental health and treatments (three questions); 2. The nature of DMHSs (three questions); and 3. Governance and eco-system (five questions). The themes, questions, and issues for consideration are shown in Table 1.

**Table 1:** Themes, questions, and issues for consideration when implementing a DMHS.

Themes	Questions	Issues for consideration
1. The nature of mental health and treatments	1.1. Which mental illness or mental health condition is the target?	√ Target condition/symptoms. √ Demographic characteristics of target population √ Severity level of symptoms √ What is out of scope
	1.2. Does the condition require treatment, assessment or information only, or a different service?	√ Will the condition benefit from DMHSs? √ What are the risks to consumers of implementation?
	1.3. What is the likely and realistic benefit of the DMHS?	√ What is the likely range of residual burden (or burden averted)? √ What does the evidence for the DMHS indicate about likely benefits (magnitude of symptom change; magnitude of improved quality of life; acceptability to consumers)? √ Will the DMHS deliver longer term services?
2. The nature of DMHSs	2.1. Is it necessary? / Why implement a DMHS?	√ What is the problem the DMHS will solve? √ Why is a DMHS a better choice than another service model? √ What will it achieve? √ What conditions will it target? √ What will it deliver? √ How will it complement and co-ordinate with existing services? √ What is the advice from stakeholders?
	2.2. What type of DMHS will be deployed	√ Specify the type and components of the DMHS √ Ensure glossary and operational definition of terms
	2.3. What will the DMHS deliver?	√ What services will be delivered (triage, assessment, treatment, onward referrals)?
3. Governance and Eco-System	3.1. Where will the DMHS fit in the broader mental health system?	√ Where will the DMHS fit with respect to other services (level of care or sequence) √ What is the relationship between the DMHS and traditional services?
	3.2. What is the funding model?	√ How will the service be funded? √ What is the length of the funding cycles and how it be extended?
	3.3. How will the DMHS integrate with other services?	√ What services will the DMHS engage with? √ What IT and system interoperability is required, how will this be funded and managed?
	3.4. How will the DMHS use emerging technologies?	√ What criteria will be used to consider emerging technologies? √ What governance frameworks/standards will be applied? √ What technical maturity is required of new technology before adoption.
	3.5. Do DMHSs require the same governance as traditional mental health services?	√ What national or international clinical standards will the DMHS adopt? √ What national or international IT security standards will the DMHS adopt?

Prefacing our eleven questions we note several points. First, in selecting questions we sought to be brief and succinct. Second, we assume everyone reading this article operates in the context of financial limitations, that policy decisions are directed by political imperatives as much as by other pressures, and that policy makers do not have specialist knowledge of mental health service provision. Third, we recognise that these questions are shaped by our specific experiences and may not be as relevant for policy makers in different healthcare systems. Fourth, we have added several observations from our work which we hope provides helpful insights. Finally, we note that policy makers are presented with advice from many stakeholders with good intentions but which represent particular viewpoints. We understand that this guide would be one of many background documents that might be considered, and any lack of detail is in the interest of brevity.

In brief, this paper was specifically written for policy makers, funders and decision makers but could also be relevant to healthcare managers who are considering adding DMHSs to existing services. It summarises several important conceptual and practical questions that should be considered before adopting DMHSs, together with recommendations based on evidence and experience. Our primary focus is on therapist, coach, or similar guided services, but our eleven questions are also relevant to self-directed or automated DMHSs. In our view, prospective consideration of these questions will increase the likelihood that the subsequently deployed DMHS models will successfully meet their objectives, be both cost and clinically effective and will generate data to inform future improvements to policy and to service models. Conversely, disregard for these questions will likely lead to wasted resources, duplication of existing services, consumer frustration and missed opportunities.

## 2 Theme 1. Questions about the nature of mental health and treatments

### 2.1 Which mental illness or mental health condition is the target?

The term ‘mental illness’, includes a range of conditions, with different etiology, symptoms, and treatments. For example, people with mild symptoms of depression and anxiety usually experience spontaneous natural remission, whereas those with moderate symptoms may benefit from short-term and low intensity psychological intervention delivered by a DMHS (Karyotaki et al., 2021). By contrast, people with severe mental illnesses such as treatment resistant major depressive disorder or schizophrenia often require long-term pharmacological and psychological treatments delivered in-person or blended with DHMS by specialist services and may require long term accommodation, vocational and other supports to optimise their recovery.

**Why is this important?** Policy makers who fail to define the target conditions risk commissioning services that are non-specific, poorly targeted, ineffective, and can delay effective treatment or even make symptoms worse. For example, people with mild symptoms usually benefit from practical advice and assurance that mild symptoms are often normal, transient, and will resolve over time, often with minor lifestyle changes, and are unlikely to require potentially dependence forming medications or intensive counselling. As the severity and complexity of symptoms increases, the need to urgently stabilise acute psychiatric symptoms takes priority over the education and training in psychological skills provided by DMHSs, although those treatments are often beneficial once the condition has stabilised. DMHSs have been shown to be helpful for mood symptoms experienced in a wide range of conditions (El Alaoui et al., 2015; Hedman et al., 2014; Hadjistavropoulos et al., 2021; Machado-Sousa et al., 2023; Mehta et al., 2018; Murray et al., 2015; Wootton et al., 2021), but remote services cannot provide urgent care to unstable and high-risk patients.

**Recommendation.** Policy makers should specify the types of mental health conditions and consumers which a DMHS policy or funding program aims to support, for example, reporting that a policy program targets adults with mild-moderate anxiety or depression in primary care. This approach will support the development of realistic expectations about the role of different services within the mental health system, while helping to identify what types of treatments and services might still be required for a comprehensive and mature mental health system. It will also help consumers develop realistic expectations about what DMHSs can and can't deliver and can serve to identify gaps in service models or areas of service duplication.

### 2.2 Does the condition require treatment, assessment or information only, or a different service?

In recent decades there has been a gradual but significant reduction in the severity of symptoms required before people receive mental health services (Frances, 2013; Olfson et al., 2019). As a result, more people than ever before now meet thresholds for receiving Government or health insurer funded treatments, leading to potential overtreatment of certain conditions at the expense of more serious and disabling conditions. While the increase in treatment coverage is beneficial for many people, the results from the World Health Organization's World Mental Health Surveys found that 15.9 % of those who received a mental health service did not meet diagnostic criteria for a mental health disorder (Bruffaerts et al., 2015). In parallel, there has been an increase in conditions that Governments and communities are concerned about, for example, social isolation (Santini et al., 2020), which although it can be a sign of a treatable disorder such as major depressive disorder, and is associated with poor quality of life, is not a mental health condition. Systems of reimbursement can encourage over-servicing of conditions which may not be amenable to treatment, at the expense of more serious mental illnesses.

**Why is this important?** Policy makers should consider the appropriate thresholds for care to mitigate at least three important risks. First, all mental health services have direct and indirect costs; for example, consumers may be prescribed psychiatric medications or psychological therapies that are not only expensive, but may be ineffective, unnecessary, or harmful in some situations. Consistent with this, Australian data showed that even when more adults were able to access mental health services, there was no discernible improvement in levels of mental health in the broader population (Jorm et al., 2017). Second, everyday life involves significant but transient disappointments and frustrations which usually resolve with time; offering treatments for such experiences may medicalise normal human discomfort and distress undermining consumers' resilience and self-efficacy and causing people to become dependent on mental health services. Third, people can become distressed for understandable reasons, including financial and relationship problems, which are often not amenable to mental health treatments and obscure the need for population-based strategies (Thangadurai and Jacob, 2014) Lund et al., n.d.).

**Recommendation.** Policy makers should consider whether a condition is likely to benefit from treatment by a DMHS, and whether the likely benefits justify any foreseeable harm. Short term improvements are observed with most treatments that involve contact with a practitioner, but policy makers should also consider whether improvements are likely to meet a minimum threshold of magnitude, whether they significantly increase quality of life, and whether the results are sustained over time. Policy makers in mental health could explore examples from other fields where the potential for overtreatment has been examined, including management of low back pain, or opioid-based pain relief. Consideration of public mental health education campaigns that aim to promote community resilience and an understanding of the differences between normal human distress and treatable symptoms should also be part of any proposed DMHS.

### 2.3 What is the likely and realistic benefit of the DMHS?

An implicit assumption across health care is that treatment resolves the target condition. Unfortunately, similar to many physical health conditions, mental health conditions are often chronic and may re-appear following treatment. Furthermore, while mental health treatments reduce the severity of symptoms there is often residual impairment or disability (Vos et al., 2004; Cross et al., 2023).

**Why is this important?** Residual disability may result in consumers requiring longer term or more intensive treatments than DMHSs can typically offer, in addition to broader psychosocial support. Most DMHSs offer a model of care that is time limited and comparatively short term, although the benefit is often long term (Andersson et al., 2017). Furthermore, by virtue of their relatively low cost, DMHSs could be used to deliver booster sessions which have a role in supporting relapse prevention particularly for consumers with chronic experiences of mental health conditions or those recovering from an episode of poor mental health and do not require a full course of treatment. An additional point to note is that not all digital psychological treatments generate equivalent clinical benefits and poorly developed treatments may increase the risk of harm.

**Recommendation.** Policy makers should recognise that no mental health treatments are perfect, that there will be residual burden and that optimising treatment requires attention to several issues. These include ensuring that the intended DMHS have a robust quantitative and qualitative evidence base, that is, they generate reliable clinical outcomes and they are acceptable to consumers. Second, optimising DMHSs involves supporting interoperability among services to facilitate referrals that would address the different areas of a consumer's life which may affect their mental health, such as access to stable housing and physical health care. This could be achieved by supporting interoperability (see below), through funding DMHSs to deliver services such as case management, or funding external case managers or system navigators (Perret et al., 2023). Third, when considering how to optimise DMHSs to deliver longer term care, policy makers will need to carefully consider how DMHSs could be used to both monitor consumers' wellbeing and deliver reminder, booster or other similar services for people with chronic symptoms and long-term needs.

## 3 Theme 2. Questions about the nature of DMHSs

### 3.1 Is a DMHS necessary?

As indicated above, DMHSs offer many potential benefits to consumers and to the broader mental health system including reducing barriers to care, providing improved access for marginalised groups, and providing clinically and cost-effective services (Andersson and Titov, 2014). However, it is important that policy makers and funders are transparent about the value proposition of DMHSs and understand the impacts of a new service on existing services, and any risks.

**Why is this important?** The introduction of new service models inevitably increases the complexity of the existing system, particularly in the case of mental health systems, where there is often limited co-ordination among services and across different levels of service. Increasing complexity will make it more challenging for consumers and referrers to understand the differences among services and how to access them. Existing mental health service providers may perceive threats to their funding and to their ability to retain staff and may not support implementation efforts, regardless of the evidence base for the DMHS. In circumstances where components of DMHSs are to be introduced into existing traditional mental health services, therapists and clinicians may also have realistic concerns about increasing volume and complexity of workloads (e.g., Van Assche et al., 2022).

**Recommendations.** Policy makers should collaborate with subject matter experts including service providers, clinicians, consumers, and other key stakeholders, to develop and transparently report the program logic of the DMHS. Descriptions of program logic achieve several important objectives including summarising the problem the service aims to address, explaining why existing service models are unable to achieve those goals, defining the mental health conditions the DMHS will help manage, and describing the types of services offered. In addition, program logic is a framework for transparently describing a service or interventions components and required resources or inputs, the proposed mechanisms of action and the expected outcomes or outputs (Centre for Epidemiology and Evidence, 2023). This description can also include the theoretical frameworks, such as health belief model (Bandura, 2001), social cognitive theory (Kelder et al., 2015) or other theories which are believed relevant (see Marcos et al., 2024 for an example). The program logic should also explain how the DMHS will complement and be coordinated with existing services as well as what it will not or cannot provide. Policy makers must then clearly communicate this information to a broad range of stakeholders, to both seek advice and feedback, but also to generate support for implementation.

### 3.2 What type of DMHS will be deployed?

Despite attempts to standardise the terminology, the terms ‘digital mental health’, ‘e-mental health’, ‘telehealth’ and ‘virtual health’ are used inconsistently, and they may refer to very different things (Smoktunowicz et al., 2020). Those terms have been variously used to refer to a type of DMHS, an electronic medical health record or other underlying technology, an online support forum or even an information website.

**Why is this important?** The risks of inconsistent or non-specific use of the term DMHS and related constructs include confusion about the targets and purpose of DMHSs and related policy. Inconsistent terminology is only likely to increase as DMHSs and related tools evolve, and as DMHSs are used more often in conjunction with in-person mental health services, that is, in so-called ‘blended care’.

**Recommendation.** Policy makers should clearly define the type and components of a proposed DMHS as well as its purpose, that is, whether it is a service, a technology platform, a combination of these, or something entirely different. For example, we now refer to the MindSpot Clinic, an Australian national DMHS, as a ‘digital psychology service’, which is a subtype of DMHSs, but which has the primary objectives of delivering psychological assessments and treatments. Given the rapid evolution of technology and services, policy documents should include a glossary and operational definition of key terms.

### 3.3 What will the DMHS deliver?

Mental health policies often assume consumers with symptoms are seeking treatment, which means that services are often organised around models of treatment. However, data from epidemiological surveys and from high volume DMHSs shows that many consumers are not necessarily seeking treatment. For example, an analysis of the World Health Organization World Mental Health Surveys comprising data from the US (Mojtabai et al., 2011) and from 24 countries (Andrade et al., 2014) reported that of people who met diagnostic criteria for a mental disorder in the previous 12 months, significant proportions did not believe they needed treatment, and a significant proportion also indicated a preference to self-manage.

Similarly, a key finding from the first eight years of operation of the MindSpot Clinic has been that fewer than 30 % of 120,000 consumers report that their primary reason for accessing MindSpot was to obtain treatment (Titov et al., 2020a), with 67 % initially reporting that their primary reason for was to obtain a confidential assessment to help them to better understand their symptoms and treatment options. Hence an important function of DMHSs may be in explaining the role of different mental health services and how to navigate the mental health system. Recent qualitative studies with MindSpot therapists and service users have confirmed that an initial online assessment is an intervention in itself, allowing consumers to decide about whether and how to seek further care (Fisher et al., 2023; Fisher et al., 2024). This does not mean that consumers do not need or do not benefit from treatment. Rather, much like physical health conditions, mental health consumers make their own decisions about whether they will engage in treatment, and what type of treatment they want, based on the information and advice provided to them.

**Why is this important?** Failure to acknowledge consumers' services preferences means that policy makers may fund services that are not used and that fail to meet consumers' needs. Furthermore, assuming that all consumers with a condition will want treatment and that treatment needs to follow the format used in traditional in-person mental health services, could lead to over-funding of services (Hadjistavropoulos et al., 2020a). Because service models will impact therapists and clinicians, these stakeholders should also be consulted to ensure service models not only meet consumers' needs but can also to be efficiently integrated into clinical workflows.

**Recommendation.** Policy makers should recognise that DMHSs, much like traditional mental health services, need to offer assessment, treatment and referral services. Policy makers should also consider how to optimise the potential of DMHSs to efficiently triage large numbers of consumers, to educate consumers about the mental health system, to explain the role of different mental health services, and to help consumers navigate the mental health and other systems and supports to find the services they need. An additional important consideration is that by virtue of their accessibility, DMHSs serve a broad cross section of the population, including marginalised communities for which there is promising evidence for some groups (e.g., Kayrouz et al., 2020) but only emerging evidence for others (e.g., Mabil-Atem et al., 2024). For this reason, where possible and sustainable, policy makers should consider how to optimise the relevance of DMHSs to diverse populations, however, it should be noted that like traditional mental health services, no single DMHS is likely to meet the needs of all possible consumers.

## 4 Theme 3. Questions about governance and eco-system

### 4.1 Where will the DMHS fit in the broader mental health system?

Mental health systems are increasingly adopting a ‘stepped care’ framework, that is, the provision of levels or ‘steps’ of mental health services based on the severity and persistence of a consumer's symptoms and needs (Bower and Gilbody, 2005; Richards et al., 2012). Within stepped-care models, DMHSs are often placed at the prevention or mild end of the spectrum of services and therefore considered a first-line service option for those with mild symptoms or for preventive interventions. However, the experiences of established and successful DMHSs do not match that neat division of responsibilities for several reasons.

First, DMHSs that offer services direct to consumers are accessed by people with all levels of severity, and even people who are severely unwell can benefit from services offered by DMHSs (Nielssen et al., 2023), providing they can understand the material and have the motivation to engage. This does not mean that DMHSs can replace more intense or higher levels of care, including pharmacological or in-patient care. Instead, DMHSs can offer immediate care to those on waiting lists (Huang et al., 2024) or as a way of delivering psychological care for those who are unable to attend in person services due to challenges relating to geographical isolation, physical disability or even because of the severity of their symptoms, or for those who prefer virtual rather in-person consultations.

Second, access to psychological treatments at the higher levels of stepped care are often limited due to workforce shortages, which means that education about symptoms and self-management skills, for which there is a good evidence base, may not be delivered in high intensity services. DMHS interventions adapted for people with more severe symptoms could be offered to complement acute care (Sawyer et al., 2023).

Third, some consumers will inevitably decline higher intensity services, despite the presence of severe symptoms, but will accept and derive benefit from low intensity services such as those provided by DMHSs with many studies showing equivalence in outcomes of face to face and internet delivered treatments (e.g, Clark et al., 2022; Hedman-Lagerlöf et al., 2023).

**Why is this important?** Careful consideration of where DMHSs fit in to stepped care or other frameworks, and some flexibility in expectation of the role of DMHSs could optimise the benefit of DMHSs to consumers. DMHSs have also played an important role outside of the traditional mental health system. For example, specialised DMHSs have been developed to support consumers in learning to manage the psychological symptoms associated with conditions such as insomnia (Blom et al., 2024; Ritterband et al., 2017; Scott et al., 2024), chronic pain and chronic health conditions (Dear et al., 2022; Lim et al., 2021), and even neurological conditions (Andersson, 2015; Gandy et al., 2023).

**Recommendations.** The question of fit has multiple layers. First, acknowledging that different health systems will have different characteristics, our recommendation is that DMHSs should be available and integrated with all levels of stepped care and potentially with other part of the healthcare system to ensure that people with a broad range of psychological conditions benefit from opportunities to obtain evidence-based treatments. Second, policy makers need to consider where a DMHS ‘fits’, and whether it sits within traditional in-person services, as a standalone service which accept referrals from a range of sources, or both? Third, policy makers and other interested parties need to consider these issues in relation to details about how personal health information will be collected, managed and shared, particularly for consumers who may not wish that their information be stored in a larger healthcare facility.

### 4.2 What is the funding model?

One of the attractions of DMHSs to policy makers is the perception that they are inexpensive to establish and operate, infinitely scalable, that the self-directed nature of interventions significantly reduces workforce requirements, and that emerging technology, such as AI, will increasingly automate components of care, further reducing costs. While DMHSs are usually provided at a low cost per patient, and are more scalable than traditional mental health services, the recurring costs of maintaining and upgrading technology platforms is not insignificant, particularly with respect to evolving IT security threats. Moreover, DMHSs that serve people with mental health conditions often require crisis support from clinically trained staff to provide a safe and responsible service (Nielssen et al., 2022).

**Why is this important?** Inadequate and short-term funding cycles impair strategic planning, and make it difficult to attract and retain staff, develop the workforce, or even lease clinical office space. Such cycles also increase the risk that the software platforms used to deliver services will eventually become unstable and unattractive, which in turn will affect the credibility of a service model and therefore engagement and outcomes.

**Recommendations.** We strongly recommend that policy makers support long-term funding models that ensure DMHSs have sufficient operating resourcing to attract and develop staff, to continue to deliver contemporary digital experiences to consumers, and to ensure that the technology base is secure. The experience from the first decades of DMHSs is that they should be funded by federal or state governments or by national insurers. To ensure high performance and high return-on-investment, we recommend that DMHSs be provided with medium-term funding agreements that automatically extend when interim performance targets are achieved. This may also require investment in a smaller number of larger DMHSs with sufficient resources to ensure adequate systems of governance, workforce development, quality assurance, evaluation, service improvement and technological development. Finally, we also recommend that in the interests of access and health equity and recognising their cost-effectiveness, DMHSs should, as much as possible, be provided at no cost to consumers. We understand that this decision needs to be based on consideration of many factors, but our experience is that the cost effectiveness of DMHSs (Lee et al., 2017) makes a compelling economic argument for such an investment.

### 4.3 How will the DMHS integrate with other services?

There is broad consensus that mental health services should work in a way that compliments other service sectors including employment, education, accommodation, welfare and other social services that influence mental health. DMHSs have also emerged in what are already complex mental health systems, funded by different tiers of government, and split between regional hospital-based services, and a wide range of private physicians, psychologists, other types of counsellors, and self-help services. Policy makers and funders sometimes assume that by virtue of being ‘digital’ that DMHSs can seamlessly integrate with other services.

**Why is this important?** Unfortunately, the successful technical and operational integration between DMHSs and other systems, that is interoperability and partnerships, is as challenging for DMHSs as for other services. Interoperability requires data systems which are secure and reliable, specialised staff with the expertise to manage the relationships and technical issues resulting from system integration, workflows which support staff to efficiently work with different services and systems, and governance frameworks which describe the roles and responsibilities of each service. Consequently, such integration is often only sustainable for large services or national organisations and in our experience even large national services rarely have efficient ways of referring consumers to other relevant services and instead often rely on the consumer to approach the other services themselves.

**Recommendation.** Policy makers should scope the types of services that the DMHS sector should engage with. Specific funding may be required to develop interoperability between data platforms and to build the interpersonal and inter-service relationships required to succeed. This requires policy makers to consider services that provide social or financial support, and how services could best be organised in the interests of consumers, noting that attention must be paid to ensuring consumers provide informed consent if data is to be shared between services (Shen et al., 2019). In addition, policy makers need to consider how the DMHS will be marketed and promoted to consumers and potential referrers such as primary care physicians who may not have familiarity of the high quality of care that DMHSs can provide. Such activities require long-term commitment and funding beyond the typical political cycle.

### 4.4 How will the DMHS use emerging technologies?

The rapid development and increasing availability of Artificial Intelligence (AI) confirms the potential of emerging technologies to increase the efficiency of DMHSs by providing services directly to consumers, providing prompts to therapists to improve their work, and by automating back-office activities, especially evaluation (Torous and Blease, 2024).

Important developments are also occurring in the use of other technologies such as virtual reality (VR) in the treatment of different disorders, particularly anxiety disorders where exposure is a key treatment component. Research into the use of VR in mental health has revealed promising results (Carl et al., 2019; Freeman et al., 2022; Rowland et al., 2022). The key challenges to implementation have included the lack of commercially available and cost-effective VR headsets and hardware required to create and deliver VR experiences. However, the use of VR in video games and virtual worlds is making such tools more accessible and therefore more likely to be used, albeit to consumers from more affluent economic backgrounds.

**Why is this important?** A key challenge for policy makers and DMHS providers is to manage the hype surrounding emerging technology, and to identify the potential risks related to IT security, consumer safety and acceptability to consumers and therapists and other stakeholders (Cross et al., 2024). DMHSs that emphasize the technological experience over mental health outcomes and proper governance, are likely to be more expensive to deliver and maintain and may risk privacy or security breaches.

**Recommendations.** Policy makers should consider how DMHSs are positioning emerging technologies and whether the new technologies are appropriate, safe and beneficial. The decision to adopt emerging technologies should be carefully informed by a business case that demonstrates the short and long-term value of adoption against relevant risks, and by reference to appropriate guidelines and governance frameworks, noting that these are also rapidly evolving. Rather than emphasising the technology itself, we recommend viewing technology as a means of increasing access to traditional mental health services or increasing efficiency. We recommend that DMHSs avoid early adoption of new technologies and instead wait for second or third generation releases which are more stable and mature.

### 4.5 Do DMHSs require the same governance as traditional mental health services?

DMHSs can be used in all levels of mental health stepped-care frameworks, including in preventive care, ‘wellbeing’ and ‘clinical services’. This means that questions can arise about whether governance frameworks, which provide oversight of a services' risks, outcomes, and quality, are always necessary.

**Why is this important?** Due to their delivery methods, governance frameworks for DMHSs need to include components concerned with both clinical and IT operations. Although this adds to the complexity and expense of services, we hold this view for at least three reasons. First, even services targeting prevention rather than clinical care will be used by consumers with severe symptoms, some of whom may be at risk of self-harm or harm to others. A clinical governance framework will reduce risk for the consumer and for the organization by defining how such consumers are identified and then supported to stay safe.

Second, IT systems require integration of different platforms such as web browsers, databases, operating systems, and AI, each of which have different risk profiles. A service delivering mental health prevention services may not aim to collect data about consumers, but software used in the delivery of prevention services such as web browsers or servers might collect IP addresses and other details which could be used to identify consumers. An IT governance framework provides a structured system for identifying and mitigating the different risks inherent in delivering any type of service using technology.

Third, robust clinical and IT governance frameworks define processes for managing technology that not only facilitates the safe delivery of DMHSs but ensures that Government, regulators, funders and the broader community have confidence in DMHSs and in the outcomes generated by the DMHSs.

**Recommendation.** It is strongly recommended that policy makers require all DMHSs, regardless of their target population, to establish and operate robust clinical and IT governance frameworks that comply with relevant quality standards. These quality standards need to include ongoing evaluation of outcomes and usage to inform quality improvement activities and future policy and practice. Governance is not static and should be regularly reviewed and updated, audited by external experts, and required to meet relevant national or international standards (for example, ACSQHC, 2020).

## 5 Discussion

The aim of the present article was to describe eleven conceptual and pragmatic questions that policy makers and funders should consider before implementing a DMHS (Table 1). These questions are more concerned with the *why* of implementation, rather than just the *how*, since there is now considerable helpful advice on how to successfully implement remote psychological care (e.g., Mohr et al., 2025).

Experience has shown that DMHSs or components of DMHSs can be placed at multiple levels within the mental health system, since there are consumers with all levels of symptom severity who are likely to benefit from education about their symptoms and information to help them to manage their own symptoms. This raises the question of whether DMHSs should be stand-alone services or added to existing services. For reasons of sustainability, acceptability, cost and clinical effectiveness it would be preferable in the long-term to add DMHSs to existing services. However, those services often do not have the capacity, capability or culture to adopt entirely new workflows and different models of care (Hadjistavropoulos et al., 2020b). Practitioners also require specialized skills working online and this requires attention to developing systems of both training and clinical supervision (Hadjistavropoulos et al., 2018; Pote et al., 2025). Given both the cultural differences between traditional and DMHSs, and the technical expertise required to deliver DMHSs, we tend to recommend the use of large scale standalone DMHSs, but we also acknowledge examples of successful services that follow a non-centralized or de-centralized model (e.g., Gervind et al., 2024).The resources required to safely and effectively operate DMHSs is another reason we prefer standalone services, particularly given the likely costs and complexity of trying to modify traditional mental health service IT infrastructure to be able to deliver components of DMHSs.

A key theme relevant to many of these eleven questions is that of expectations of DMHSs. We described the reality that no mental health service, delivered either traditionally in-person or digitally, ‘cures’ people. We also noted that by virtue of using technology, DMHSs are subject to unrealistic expectations including that they may be seen to be the panacea to address all inequalities in society. Although high volume DMHSs can improve access to evidence-based care, it is not necessarily those with the least access to care who make the most use of DMHSs.

A theme common to many of our questions is the complexity of DMHSs and the requirement for a highly competent clinical and non-clinical workforce. Lack of engagement and support from the clinical workforce is a well-recognised risk factor to the successful implementation of DMHSs (Gaebel et al., 2021; Van Assche et al., 2022). This complexity is only likely to increase as new technologies such as AI become integrated into models of care and support systems.

Several of the questions posed, including the target condition or disorder, and whether the condition requires mental health treatment or a different service, are relevant to the broader mental health system. These questions have enormous implications for funding and for the structure and scope of mental health services. Naturally, the answers will vary depending on the target population, the availability of funding, and political will. Politicians are typically motivated to address the needs as expressed by the community or at least the most vocal parts of the community for more services and tend to have a shorter-term view than is always beneficial. Denying requests for more and more services is politically difficult, but the reality is that no health system has the financial investment or workforce to meet all the perceived needs. Moreover, some conditions cannot be adequately treated by DMHSs or traditional mental health services and sometimes should not be.

A key recommendation of this article is that DMHSs should, where possible, be provided at no or low cost to consumers in the interests of access and health equity. There are different ways to achieve this outcome, either by block funding or through universal health care providers, but our experience is that many of the >300,000 Australians who have accessed MindSpot were unable to access traditional mental health care due to barriers that include remote location, cost, stigma, limited and access to clinicians (Titov et al., 2020a). The relatively low cost of providing assessments and effective evidence-based care via a national DMHS, and the clear functional improvements resulting from their treatment provides a compelling economic argument.

An overarching guiding principle which may support policy and decision makers in navigating these eleven questions and formulating *how*, *where* and *why* they will implement DMHSs into their mental health systems should be the requirement for a clear statement of program logic. A statement of goals can explain the gap the proposed DMHS seeks to address, the way it will work to achieve defined goals, the inputs and resources required, the expected outputs and outcomes, and how risks will be managed. We encourage policy and decision makers to transparently share their program logic with key stakeholders in order to maximise support during implementation. We also hope the details in Table 1, including the issues for consideration will assist policy makers and funders in their consideration of DMHSs.

Finally, we recognise that digital mental health care and the surrounding social and technological context is evolving rapidly, posing poses challenges for how policy makers and funders, as well as service providers, navigate uncertainty and make optimal strategic and operational decisions. As a complement to the statement of program logic we would encourage policy makers to also explicitly define decision-making principles for adopting and planning DMHSs. Although each jurisdiction will have their own specific principles and priorities including those related to efficiency, equity and acceptability, we would encourage the inclusion of a requirement for evidence of clinical and cost-effectiveness and safety before adoption. This applies to emerging technologies and applications of artificial intelligence, but also applies to other potential new treatment models. Such a framework will help balance the need to demonstrate a commitment to innovation with the responsibility of ensuring services are successful in achieving the relevant healthcare goals.

We acknowledge several important limitations to this work. The eleven questions considered here were selected based on the authors' experiences working with policy makers and funders in particular contexts. We recognise that even within culturally similar countries and even within countries healthcare systems are diverse and our questions and experiences may not be relevant in other contexts. We also recognise that there are many other questions that can be considered, such as questions related to workforce planning and measurement of outcomes, though we suggest these may be more relevant to *how* DMHSs are implemented rather than *why* they are implemented.

## 6 Conclusions

DMHSs are coming of age. There is incontrovertible evidence of effectiveness and efficiency, and given the scale of the problem, DMHSs will be an essential component of attempts to address the unmet need for evidence-based care. As with any endeavour, careful planning significantly increases the chance of successfully implementing DMHSs. The eleven questions and recommendations were proposed to support policy makers and funders to consider *why* they are considering implementing DMHSs, with the aim of increasing their likely success, based on our experience implementing DMHSs in several countries. We emphasize the importance of realistic expectations, stable funding and clear program logic. However, just as technology evolves, so too will the key questions. In summary, our experience with DMHSs is that although they are not a panacea, they have enormous potential for improving access to evidence-based mental health services. We stress that their success is dependent on realistic and practical mental health policies and long-term funding.

## Funding

This article was supported by caffeinated products.

## Declaration of competing interest

All authors are involved in developing and delivering digital mental health services. The authors have no other disclosures.
